# Why implementation gaps could undermine synthetic nucleic acid oversight

**DOI:** 10.3389/fbioe.2025.1689753

**Published:** 2025-10-28

**Authors:** David R. Gillum, Rebecca L. Moritz

**Affiliations:** ^1^ Compliance and Research Innovation, University of Nevada, Reno, NV, United States; ^2^ Tutela Strategies, LLC, Reno, NV, United States

**Keywords:** synthetic nucleic acids, biosecurity policy, sequence screening, implementation gap, institutional capacity

## Abstract

**Introduction:**

Recent U.S. biosecurity policy has shifted from organism-level controls to sequence-level governance of synthetic nucleic acids in response to *de novo* genome synthesis risks, artificial intelligence assisted design, and globalized DNA/RNA manufacturing. While intended to strengthen safety and security, this shift risks overburdening under-resourced institutions and providing oversight that looks thorough on paper but delivers little added protection. This study examines the widening “implementation gap” between policy ambition and operational capacity.

**Methods:**

Drawing on practitioner experience and current literature, we analyzed policy frameworks, institutional practices, and case examples to identify structural challenges in sequence-level oversight. Particular attention was given to how definitions, regulatory triggers, and institutional resources interact in practice, creating gaps between policy intent and operational capacity. This mixed approach allowed us to capture the high-level design of oversight frameworks and the practical realities of their implementation across diverse institutional settings.

**Results:**

We found three core obstacles: ambiguous definitions of sequences of concern, fragmented and overlapping regulatory triggers, and underdeveloped institutional screening and review capacities. Ambiguity creates uncertainty about what should be flagged, while fragmented rules add redundancies without clarifying responsibility. Limited institutional resources further constrain effective oversight. These weaknesses produce overinclusive surveillance, inconsistent provider screening, unmanaged legacy construct inventories, and a lack of shared reference tools, straining resources without yielding proportional security benefits.

**Discussion:**

Aligning oversight with real-world capacity is essential to avoid brittle and costly systems that deliver limited biosecurity benefits. We propose seven reforms to address the identified obstacles: functional risk tiering, federal investment in biosafety infrastructure, policy pilots and real-world testing, institutional certification pathways, adaptive governance cycles, pragmatic global harmonization, and coupling screening with operational safeguards. These measures reduce ambiguity, streamline fragmented rules, and strengthen institutional capabilities. Embedding implementer perspectives and calibrating oversight to realistic capacities will ensure that biosecurity systems remain credible, resilient, and effective in the synthetic nucleic acid era.

## 1 Introduction

Internationally, efforts like the International Biosecurity and Biosafety Initiative for Science (IBBIS) “Common Mechanism” aim to offer a shared, open baseline for synthesis screening, acknowledging both provider constraints and the need for global consistency (Wheeler et al., 2024a; EBRC, 2024). However, adoption requires trust, resources, and integration across heterogeneous institutional systems, challenges that are magnified in academic settings (Wheeler et al., 2024a).

A recent development shaping genetic screening expectations for U.S. institutions was the *2024 Framework for Nucleic Acid Synthesis Screening* (OSTP, 2024; ASPR/HHS, 2024; later rescinded by Executive Order 14292 in 2025). This framework recommended that providers screen orders for sequences of concern (SoCs), verify customer legitimacy, maintain transaction records, and adhere to cybersecurity standards and was built on previous guidance (DHHS, 2010). The National Institute of Standards and Technology (NIST) was tasked with developing the supporting technical standards (NIST, 2024).

At a high level, these actions appear logical: screening sequences, verifying customers, and mitigating misuse risks. However, in practice, the repercussions are significant. Few entities have (i) institution-wide sequence screening capability, (ii) trained biosecurity reviewers, and (iii) resources to inventory and risk-assess potentially tens of thousands of legacy constructs in refrigerators and freezers. Published research shows that many biosafety offices operate with only a handful of staff, acknowledging a challenge in this space (Gillum et al., 2024b).

During the past 18 months, U.S. government initiatives have expanded the scope and intensity of oversight across synthetic nucleic acids, dual-use research, and so-called “dangerous gain-of-function (dGOF)” experiments (OSTP, 2024; White House, 2025). The intent is to reduce the chance that cutting-edge biotechnology could be misused. However, *intent* does not include *implementation*. The body of literature documents the expanding expectations around synthesis screening, highlights unresolved implementation questions, and describes variability across providers, which raises concerns about feasibility and consistent risk reduction in practice (Carter et al., 2024a; Carter et al., 2024b; Rose et al., 2024; Wheeler et al., 2024a; Kane and Parker, 2024).

The shift is not simply about increasing oversight; it represents a structural reorientation from organism-level control (e.g., Select Agent lists, export control lists, risk group classifications) to governance of specific genetic sequences regardless of the host or system (including cell-free platforms) in which they are expressed. This pivot is defended as closing gaps exposed by *de novo* synthesis, AI-assisted design, and the globalization of DNA/RNA manufacturing (Sharkey et al., 2024; Wheeler et al., 2024a; Batalis et al., 2024). At the same time, it creates expansive gray zones where benign genetic fragments and routine constructs may be swept under “Sequences of Concern,” even as the practical contribution to threat reduction remains uncertain (Godbold and Scholz, 2024; Gemler et al., 2024).

We write from the perspective of implementers. Throughout our careers, we have translated government policy into practice, built compliance systems, and advised regulatory agencies. We now witness a growing disconnect between policy goals and institutional reality. This paper examines why the current trajectory risks producing a compliance system that is brittle, costly, and under certain circumstances symbolic rather than substantive implementation. Addressing these gaps will require sustained investment, realistic scoping, and genuine collaboration with the people who must implement and manage the systems.

## 2 Why basic constructs should not be overregulated

Viral infection begins with attachment to a host cell, a prerequisite for entry, replication, and pathogenesis. Lacking their own metabolic and biosynthetic machinery, viruses depend entirely on their hosts for reproduction. Therefore, understanding this attachment process is fundamental to understanding viral disease mechanisms (Jackson et al., 2022). Fortunately, studying the proteins responsible for cell binding does not require using a live, fully infectious virus. Safer *in vitro* systems, such as plasmid-based expression in cell culture, pseudotyped viruses, and virus-like particles, can model key aspects of viral entry and provide valuable insights (Takada et al., 1997; Schmidt et al., 2020; Rizatdinova et al., 2025). For example, the Ebola virus glycoprotein (GP) is widely studied using non-infectious, non-replicating plasmid constructs, which enables researchers to investigate receptor binding and membrane fusion without handling the pathogenic virus (Steeds et al., 2020).

These tools are broadly recognized as safe and indispensable for dissecting host-pathogen interactions. However, under future U.S. biosecurity policy, even these benign constructs may require screening, additional institutional review, and external oversight, which could potentially burden routine science and additional administrative oversight. This concern is concrete rather than hypothetical, as it would immediately impact widely used constructs with minimal risk profiles. Genetic elements, such as receptor binding mutants, protective antigen domains, or plant virus proteins, are frequently used in benign, well-established research contexts. If categorized too broadly as SoCs, they risk triggering oversight that far outweighs their minimal hazards (Rose et al., 2024; Wheeler et al., 2024a).

## 3 Sequence screening risks and tradeoffs

The moral imperative behind sequence screening is straightforward: do not sell dangerous biological components to those who might misuse them. However, the underlying threat model is often implicit, loosely specified, or extrapolated from worst-case scenarios (Dieuliis et al., 2024). The key questions are therefore not only technical but also fundamentally political: who should be allowed to use what, and under what conditions? In practice, these requirements cascade to universities and other research institutions, where decentralized procurement systems and long histories of working with legacy constructs mean compliance falls unevenly on institutions and even individual laboratories.

First, screening seeks to block many of the capabilities that can also be achieved through established microbiological methods, such as polymerase chain reaction (PCR) amplification from environmental samples, cloning from readily available strains, or reassembling published sequences. This is particularly true for gene fragments or widely distributed functional elements. While screening may raise the time or financial cost for some actors, it is unlikely to impose a meaningful barrier to a motivated and skilled adversary (Batalis et al., 2024).

Second, emerging technologies such as large language models (LLMs) and advanced biodesign tools do present dual-use potential. They may reduce certain planning burdens or facilitate the generation of novel sequences. However, empirical studies show that translating an *in silico* design into a functional organism requires substantial laboratory infrastructure, tacit expertise, and iterative experimentation (Batalis et al., 2024; De Haro, 2024). The risks associated with AI are tangible, but their marginal contribution compared with long-standing wet-lab routes must be weighed carefully to avoid overemphasizing the newest perceived threat.

Third, a focus on sequence-based controls risks diverting attention from operational safeguards that may yield more tangible security benefits. These include robust training programs, a strong culture of incident reporting, laboratory access controls, biological inventory management, and genetic biocontainment strategies for engineered organisms (Payne et al., 2024).

In short, screening is a valuable tool but not a panacea. Without a clear articulation of the specific adversaries, plausible misuse scenarios, and measurable risk reduction it delivers, there is a risk of constructing a burdensome compliance system for researchers while delivering only modest security gains. The real challenge is to calibrate screening alongside complementary safeguards that maximize both security and scientific progress (Rose et al., 2024).

## 4 The regulatory cascade

A significant concern for institutions will be determining which SoCs already possessed in laboratories are covered by the new gene synthesis screening requirements. In addition, core facilities that generate genetic sequences will be required to screen customers, something they are ill-equipped to do in an academic setting. Where industry has moved toward coordinated norms (e.g., the International Gene Synthesis Consortium [IGSC] Harmonized Screening Protocol v3.0), academic institutions sit at a complex convergence point: multiple funders, decentralized laboratories, open science incentives, and thinly staffed compliance offices (IGSC, 2024; Kane and Parker, 2024).

The move from organism-level to sequence-level oversight is arguably the most consequential shift of this policy moment. SoC-based control seeks to capture gene fragments or motifs (i.e., short, recurring genetic patterns associated with specific biological functions) that contribute to pathogenicity or toxicity, even outside the genomic context of a regulated pathogen (Godbold and Scholz, 2024). Proponents of this shift emphasize perceived risks: modern synthesis can assemble complete viral genomes from constituent parts; short oligonucleotides may encode peptides with functional consequences; and AI-guided design could, in theory, produce novel, unlisted variants (Sharkey et al., 2024; Batalis et al., 2024; De Haro, 2024). While these scenarios drive much of the concern, the technical barriers, biosafety measures, and institutional controls in place mean that most such sequences pose minimal hazard, particularly when used in research laboratories. Many of these genetic fragments would require additional complementary sequences, specific host factors, and specific experimental conditions to present any realistic threat. Therefore, the challenge, and opportunity, for a new policy is to develop oversight that is evidence-based risks rather than to worst-case hypotheticals.

Developing effective oversight is challenging when genetic material central to legitimate science can be misclassified as inherently dangerous. Overly broad categorization of benign constructs risks diverting attention and resources from safeguards that offer tangible security benefits, consuming time and effort without yielding proportional gains in biosafety or biosecurity. In order to cause harm, an actor would need to obtain all necessary portions of the genome essential to form a functional organism that could mimic or replicate the original. Obtaining a sample from nature using traditional microbiology techniques may often be simpler and less visible than navigating regulated synthesis routes. The challenge is compounded by ambiguity and database gaps: virulence-factor labels often conflate pathogenesis-relevant and housekeeping roles, while many functionally concerning sequences are inconsistently annotated across repositories (Godbold and Scholz, 2024; Wheeler et al., 2024a). Sensitivity studies show small interpretive changes can dramatically alter what gets flagged, amplifying inconsistency and workload (Gemler et al., 2024).

The cumulative effect is an increase in oversight requirements without a corresponding increase in actionable security signals. In a typical research laboratory, the historical constructs, many created years earlier, runs to the tens of thousands. Many of these historical constructs have changed hands due to sharing with collaborators or through requests. Auditing, classifying, and tracking these against evolving SoC lists is a nontrivial, resource-intensive task (Rose et al., 2024; Carter et al., 2024a; Kane and Parker, 2024).

## 5 The illusion of enforceability

Policies often presumes institutions have the people, processes, and platforms to execute. Most do not. University biosafety offices commonly operate with fewer than three full-time professional staff who must simultaneously cover Institutional Biosafety Committees (IBCs), Dual Use Research of Concern (DURC) reviews, Dangerous Gain-of-Function (dGOF) reviews, chemical safety, Institutional Animal Care and Use Committee (IACUC) support, incident response, training, and other mandated responsibilities (Gillum et al., 2024b). Few possess commercial screening software for SoCs; fewer can perform entity-wide sequence audits or maintain validated SoC reference sets (Rose et al., 2024; Kane and Parker, 2024). Oversight is especially thin around off boarding challenges, such as tracking synthetic DNA stocks when a laboratory closes or a project ends, and ensuring responsibility for custody or destruction is clearly assigned.

A common mitigation strategy is intercepting potentially hazardous materials at the point of procurement. While this approach can effectively screen new orders, it does not address legacy collections, materials provided by collaborators, or sequences generated on benchtop DNA/RNA synthesis instruments, which are becoming increasingly accessible in academic and even community laboratories (Adam and McArthur, 2024). Institutional procurement processes are often complex, and the widespread use of purchasing cards (i.e., institutional credit cards) introduces additional challenges. Such cards typically bypass pre-approval requirements, limiting oversight of acquisitions. In addition, tight laboratory budgets often push investigators toward the lowest-cost vendors, regardless of whether those providers meet recognized screening or compliance standards.

Substitution or workflow attacks on instruments (e.g., swapping nucleotide inputs to yield a different molecule than the screened digital design) illustrate how SoC screening alone cannot secure the full “design-to-DNA” pipeline (Adam and McArthur, 2024). In addition, procurement of synthetic genomic elements can be done centrally or through the use of purchase cards by individuals. Importantly, to date no confirmed malicious incidents involving synthetic nucleic acids have been documented, demonstrating that current policy often responds to hypothetical rather than empirical threat models.

Even the manufacturing side is heterogeneous. Kane and Parker’s interviews with providers found substantial variation in sequence screening sensitivity, oligo treatment, monitoring/evaluation, and law-enforcement reporting protocols despite broad adherence to IGSC expectations (Kane and Parker, 2024). Screening thus remains a fragmented patchwork, marked by “gaps by design” (e.g., exclusion of short oligonucleotides from screening) and “gaps by variation” (e.g., differences in provider pipelines, databases, and thresholds). For implementers, this heterogeneity translates into uncertainty: was a given construct “screened” in a way that aligns with our risk posture, jurisdiction, and obligations?

Without institutional support, compliance risks become largely performative. Policies are satisfied on paper, emails are filed, attestations are signed, procurement holds are logged, and underlying hazards may remain insufficiently addressed. Scarce time and expertise are diverted from higher-value risk controls such as training, incident response, and culture building.

## 6 Evidence from the trenches

Recent implementation research and field interviews reveal persistent and interrelated gaps in how sequence oversight is applied in practice (Gillum, 2025). Many institutions still lack fully functional Institutional Review Entities (IREs), the bodies tasked with provide multidisciplinary oversight for potential research misuse, with the breadth of expertise necessary to evaluate biosecurity issues, creating bottlenecks and variability in review quality (Rose et al., 2024). The policy environment compounds these challenges with overlapping rules that create uncertainty about triggers, thresholds, and reporting lines. This confusion produces defensive over-flagging (where benign constructs are flagged to avoid liability) in some cases and selective avoidance (where potentially relevant materials or discussions are deliberately sidestepped) in others (Carter et al., 2024a; Rose et al., 2024). The result is a climate in which individuals often steer clear of high-risk research domains and avoid public discussion, fearing audits, misinterpretation, or reputational damage (Kane and Parker, 2024; Gillum, 2025).

These oversight challenges, organizational differences, and resource limitations are often underestimated in policy discussions. Researchers have been ordering synthetic nucleic acids for well over three decades, driven by needs that range from cloning genes for basic research to constructing expression systems for protein production to designing probes, primers, and synthetic controls for diagnostics. Over this time, enormous quantities of oligos, plasmids, gene fragments, and synthetic constructs have accumulated in laboratories. Many of these items are freely exchanged among collaborators, transferred through informal networks, or accessed via repositories such as Addgene. In practice, obtaining a plasmid from a colleague is often quicker and cheaper than ordering or making a new one, especially when research budgets are constrained. As funding tightens, researchers may be increasingly inclined to source materials from existing stocks rather than purchase newly synthesized sequences. This means that “legacy” materials, which may encode virulence factors, toxins, or other sequences, are now under heightened scrutiny but remain widely distributed across freezers and repositories, often with minimal documentation or administrative controls compared to newly synthesized orders.

At the same time, the absence of authoritative, accessible SoC reference databases or validated screening test suites forces institutions and providers to rely on *ad hoc* lists and heuristics, virtually guaranteeing inconsistency (Wheeler et al., 2024b; Gemler et al., 2024). Encouragingly, work to develop standardized test sets and baseline criteria is underway. Wheeler et al. (2024b) describe a prototype screening dataset designed to benchmark performance and clarify regulatory ambiguities. Rose et al. (2024) outline practical questions that must be resolved to make customer and sequence screening viable in academic and commercial contexts. These prototypes represent the early stages of a necessary but overdue shift from abstract policy mandates to operational, testable, and measurable capabilities.

## 7 The cost of mistrust

Behind technical and procedural challenges lies a cultural one: mistrust. Policies are often drafted far from laboratories; guidance and companion tools arrive late; and institutional practitioners are seldom included meaningfully in the design phase. Implementers come to view policy as out of touch and overly burdensome. While policymakers see institutions as slow or reluctant. The result is a feedback loop: poor implementation → more prescriptive policy → higher burden → more disengagement. This dynamic is not unique to biosecurity. Similar cycles are documented in public health, cybersecurity, and education policy (Coburn, 2001; Strehlenert et al., 2019; Kashef et al., 2023).

Yet, these dynamics run counter to evidence on effective governance. When implementers have been integrated early (e.g., tailoring exemptions or streamlined routes for time-critical diagnostics, or piloting oversight changes with a small, diverse set of institutions), outcomes have been more balanced, adoption smoother, and compliance more authentic. For example, the most tangible progress in synthesis screening has come from industry-policy co-development (IGSC protocols; IBBIS Common Mechanism) rather than unilateral mandates (Wheeler et al., 2024a; IGSC, 2024).

Oversight of synthetic nucleic acids has often defaulted to treating all SoCs as equivalent, regardless of their functional ability. Clarifying the distinction between current requirements (e.g., provider compliance tied to federal funding in the 2024 OSTP Framework and Section 4(b) of EO 14292) and potential future oversight helps ensure that both policymakers and practitioners share a realistic understanding of what is in place versus what may still be under consideration. The current approach risks expending scarce institutional and regulatory resources on benign or ubiquitous sequences while overlooking constructs that pose genuine misuse potential. The next-generation of biosecurity governance should move beyond static sequence surveillance toward functional risk tiering that prioritizes oversight for genetic constructs whose biological activity, assembly ability, and plausible accessibility make them significant in threat scenarios. Doing so requires distilling scientific insight, operational practicality, and global coordination to ensure that policy is both risk-proportionate and implementation-ready. Here are several recommendations.

1. Shift from sequence surveillance to functional risk tiering (see DiEuliis et al., 2024; Wheeler et al., 2024b for related frameworks). Not all sequences are equal. Oversight should prioritize constructs with plausible misuse potential in context: whole-genome synthesis of high-consequence agents; recombinants or mutants restoring or enhancing pathogen function; multi-gene modules that plausibly enable or increase virulence or toxicity. Harmless fragments and ubiquitous motifs should not trigger burdensome review. This functional tiering should be grounded in transparent, continuously updated ontologies and annotations (Godbold and Scholz, 2024), and supported by empirical screening test sets (Wheeler et al., 2024b) and sensitivity benchmarks (Gemler et al., 2024).
2. Fund a national biosafety and biosecurity infrastructure initiative. Mandates without resources are performative. A national biosafety and biosecurity agency could provide federal investment by supporting (i) staffing grants for biosafety and biosecurity roles; (ii) shared tools (screening software licenses, SoC reference databases, automated audit support); (iii) training programs and communities of practice; and (iv) national centers of excellence in implementation science for biosecurity (Gillum et al., 2024a). NIST’s role in standards is welcome but must be complemented by resourcing for the people who will use those standards (NIST, 2024).
3. Mandate policy pilots and real-world testing. Before nationwide rollout, any new oversight system should be piloted with diverse institutions (e.g., Carnegie R1 universities, medical centers), collecting metrics on feasibility, burden, timelines, and unintended effects. Drawing on lessons from cybersecurity, red-teaming, and vulnerability disclosure for screening pipelines should be normalized but also conducted responsibly to avoid creating new risks or forcing rushed patches (Millett, 2024).
4. Establish institutional certification and maturity pathways. Create a biosafety maturity model with certification tiers. Institutions that demonstrate robust governance, training, incident reporting cultures, and validated screening/workflow controls should be permitted streamlined oversight (e.g., delegated approvals, reduced frequency of external reporting). This aligns accountability with capability and encourages investment in capacity.
5. Build adaptive governance with built-in review cycles. Every major policy should include 3–5-year reviews with explicit triggers for amendment, keyed to (i) implementer feedback; (ii) performance metrics and near-miss/incident data; and (iii) technology shifts (e.g., benchtop synthesizers, AI tools). Adaptive governance should evaluate risk reduction, burden-to-benefit ratios, and opportunity costs to legitimate research (Dieuliis et al., 2024).
6. Harmonize globally, baseline pragmatically. Given the borderless nature of sequence orders and collaboration, unilateral national rules produce leakage and “provider shopping.” Backing standard global baselines (e.g., IBBIS Common Mechanism; IGSC protocols) can raise the minimum while allowing local tailoring. Practical test suites and transparent update processes will build trust (Wheeler et al., 2024a; Kane and Parker, 2024).
7. Pair screening with operational safeguards. For engineered microbes, genetic biocontainment can reduce reliance on administrative controls (Payne et al., 2024). For device-based synthesis, secure-by-design features, tamper-evident consumables, and end-to-end verification (design → build → verify) address threats like substitution attacks (Adam and McArthur, 2024).

Transitioning from a purely sequence-based model to a functional, context-aware framework is essential for aligning biosecurity oversight with real-world risk. Such a shift demands more than policy language: it requires funding the infrastructure, expertise, and tools needed for sustained implementation; piloting and stress-testing oversight mechanisms before scale-up; and rewarding institutions that invest in robust oversight. Equally important is building adaptive, globally harmonized systems that evolve with technology and empirical evidence, pairing screening with practical safeguards at synthesis and in experiments. By embedding continuous review, transparency, and proportionality into governance, the life sciences community can strengthen security without stifling legitimate research, ensuring that oversight remains credible.

## 8 Conclusion

The danger of over-regulation is not a curiosity. It is emblematic of a broader problem: a governance model that risks labeling routine, low-risk science as presumptively dangerous, while under-resourcing the people responsible for actual oversight and risk mitigation. Most genetic engineering tools are harmless in common research applications, but, like almost everything in a laboratory, they can pose risks if misused. It is unclear if the burdens posed by new Federal genetic sequence screening policies will be justified. What will decide the future of biosecurity is not the sophistication or quantity of policy, but the implementation, shaped by the daily work of biosafety officers, review committees, lab managers, and scientists. Just as with the Ebola GP fragment, the danger is not in the sequence itself, but in the mismatch between how science works and policy ambition.

Rebuilding from the middle means shifting from control to collaboration, and from mandates to shared visioning. Biosafety professionals are not obstacles to national security; they are its foundation. If their insight, buy-in, and capacity are sidelined, even the best-written policy becomes another burden: fragile, symbolic, and ultimately ineffective.

We face a choice. Double down on a surveillance-heavy model that institutions cannot realistically enforce or pause, pilot, invest, and co-produce a system that is both meaningful and manageable. Only the latter path leads to resilience. Policymakers should, within the next 12 months, fund and launch a national pilot across diverse institutions to test functional risk tiering and shared screening tools, building an oversight system that is proportionate, practical, and trusted.

## Data Availability

The original contributions presented in the study are included in the article/supplementary material, further inquiries can be directed to the corresponding author.
